# Paternity through use of assisted reproduction technology in male adult and childhood cancer survivors: a nationwide register study

**DOI:** 10.1093/humrep/dead026

**Published:** 2023-02-11

**Authors:** Michael Kitlinski, Aleksander Giwercman, Angel Elenkov

**Affiliations:** Faculty of Medicine, Medical University of Gdańsk, Gdańsk, Poland; Department of Translational Medicine, Clinical Research Centre, Lund University, Malmö, Sweden; Department of Translational Medicine, Clinical Research Centre, Lund University, Malmö, Sweden; Reproductive Medicine Centre, Skåne University Hospital, Malmö, Sweden; Department of Translational Medicine, Clinical Research Centre, Lund University, Malmö, Sweden; Reproductive Medicine Centre, Skåne University Hospital, Malmö, Sweden

**Keywords:** male cancer survivors, assisted reproduction, IVF, ICSI, male infertility

## Abstract

**STUDY QUESTION:**

How does a history of cancer affect the likelihood of using assisted reproduction in order to achieve paternity?

**SUMMARY ANSWER:**

As compared to men with no history of cancer, use of assisted reproduction to achieve paternity was more frequent in fathers with a history of cancer, mainly those with testicular, prostate, and hematological and lymphatic malignancies.

**WHAT IS KNOWN ALREADY:**

Although it is well known that different types of cancer and their treatment may have a negative impact on fertility, there is a lack of data regarding the use of IVF and ICSI among male cancer survivors.

**STUDY DESIGN, SIZE, DURATION:**

In this population-based nation-wide study using the Swedish Medical Birth Register, we identified all men who fathered their first-born child in Sweden between 1994 and 2014. Using personal identification numbers, anonymized data from the Swedish National Quality of Assisted Reproduction Register, Swedish Cancer Register, Swedish Multi-generation Register, and Swedish Education Register were linked with the Swedish Medical Birth Register.

**PARTICIPANTS/MATERIALS, SETTING, METHODS:**

During the study period, a total of 1 181 488 men fathering their first-born child were identified. Of these, 26 901 fathers had a cancer diagnosis. Fathers diagnosed with cancer with <12 months from offspring conception, or with a cancer diagnosis after offspring conception, were excluded (n = 21 529). The remaining fathers who had a history of cancer (n = 5372) were divided into three groups based on age at cancer diagnosis (<15, ≥15 and <24, or ≥24 years). For subgroup analyses, they were also grouped according to the cancer location using ICD-7 codes. The fathers with no cancer diagnosis (n = 1 154 587), were included as controls. In total, 1 159 959 men were included. Associations between IVF/ICSI use and history of cancer were evaluated using logistic regression models, unadjusted and adjusted for paternal education, fathers age at childbirth, and year of conception, yielding crude and adjusted odds ratio (aOR), respectively, with a 95% CI.

**MAIN RESULTS AND THE ROLE OF CHANCE:**

As compared to controls, childhood cancer survivors were only more likely to achieve paternity through ICSI (aOR 3.52, 95% CI 2.52–4.93; *P* < 0.001) but not through IVF treatment (aOR 1.02, 95% CI 0.61–1.70; *P* = 0.955). Similarly, teenage and young adult cancer survivors were more likely to father through ICSI treatment (aOR 6.84, 95% CI 5.64–8.30; *P* < 0.001) but not using IVF (aOR 1.27, 95% CI 0.90–1.80; *P* = 0.17). However, adult cancer survivors were more likely to conceive through either ICSI (aOR 5.52, 95% CI 4.86–6.27; *P* < 0.001) or IVF treatment (aOR 1.32, 95% CI 1.09–1.60; *P* = 0.004). In subgroup analyses, childhood survivors of testicular cancer (aOR 5.15, 95% CI 1.20–22.0; *P* = 0.027), soft tissue and bone cancers (aOR 4.70, 2.13–10.4; *P* < 0.001), hematological and lymphatic cancers (aOR 4.49, 95% CI 2.72–7.40; *P* < 0.001), or central nervous system (CNS) and eye cancers (aOR 2.64, 95% CI 1.23–5.67; *P* = 0.012), were at an increased likelihood of fathering through ICSI. Teenage and young adult survivors of testicular cancer (aOR 15.4, 95% CI 11.5–20.7; *P* < 0.001), hematological and lymphatic cancers (aOR 9.84, 95% CI 6.93–14.0; *P* < 0.001), or soft tissue and bone cancers (aOR 6.83, 95% CI 3.53–13.2; *P* < 0.001) were more likely to father through ICSI treatment. Adult survivors of prostate cancer (aOR 15.7, 95% CI 6.70–36.9; *P* < 0.001), testicular cancer (aOR 9.54, 95% CI 7.81–11.7; *P* < 0.001), hematological and lymphatic cancers (aOR 11.3, 95% CI 8.63–14.9; *P* < 0.001), digestive, respiratory, and urogenital tract cancers (aOR 2.62, 95% CI 1.75–3.92; *P* < 0.001), CNS and eye cancers (aOR 2.74, 95% CI 1.48–5.08; *P* = 0.001), or skin cancer (aOR 1.68, 95% CI 1.08–2.62; *P* = 0.022) were more likely to father through ICSI treatment. Only teenage and young adult survivors of hematological and lymphatic cancers (aOR 1.98, 95% CI 1.10–3.56; *P* = 0.022) and adult survivors of testicular cancer (aOR 1.88, 95% CI 1.37–2.58; *P* < 0.001) were significantly more likely to achieve fatherhood using IVF treatment.

**LIMITATIONS, REASONS FOR CAUTION:**

Information on men failing to father children was not available, and thus our results cannot estimate the risk of infertility in men with a history of cancer.

**WIDER IMPLICATIONS OF THE FINDINGS:**

Use of ART, in particular ICSI, was significantly more frequent in fathers with malignancies of the male reproductive tract or hematological and lymphatic systems. Our findings highlight which groups of male cancer survivors would benefit from access to fertility care, thereby improving future fertility treatment policies.

**STUDY FUNDING/COMPETING INTEREST(S):**

The study received funding from the Swedish Cancer Society, Swedish Childhood Cancer Society, and the Swedish Government Fund for Clinical Research. There are no competing interest.

**TRIAL REGISTRATION NUMBER:**

N/A.

## Introduction

It is well known that male cancer survivors are more likely to suffer from infertility than other males (Magelssen *et al.*, 2006). This is mainly due to utilization of various surgical procedures and cytostatic and radiotherapy regimens which can have a negative impact on semen quality, by impairing ejaculation or hypothalamic, pituitary, and/or testicular function (Green *et al.*, 2010). Over the last few decades, major advances in the field of oncology have led to average 10-year survival rate reaching nearly 80% in patients with childhood, adolescence, or young adulthood cancer (Smith *et al.*, 2010). With a globally growing population of young male cancer survivors, more patients are expected to experience reduced fertility. For men with impaired semen quality following cancer treatment (Thomson *et al.*, 2002; Delessard *et al.*, 2020; Quera *et al.*, 2021), the most obvious way of achieving fatherhood would be by using ART, such as IVF or ICSI. Conception through IVF is most common in cases of less severe forms of male subfertility, impaired female fertility, or a combination of both. ICSI treatment, on the other hand, is recommended for men with more severe impairment of fertility (Hamberger *et al.*, 1998; Palermo *et al.*, 2015). It is estimated that in 2013, up to 3.6% of offspring born in Sweden were conceived through ART (‘Resultat-trender-öppna jämförelser Årsrapport 2015 gäller behandlingar utförda 2013’, 2015).

Multiple studies have analyzed the use of fertility preservation and outcomes of assisted reproduction in men with history of cancer (Ferrari *et al.*, 2016; Papler *et al.*, 2021; Stigliani *et al.*, 2021). However, studies exploring the paternity rates through ART after cancer treatment and identifying subgroups of male cancer survivors having the highest proportion of children conceived by ARTs are few (Gunnes et al., 2016; Bandak *et al.*, 2022), and no analysis discriminating between use of IVF and ICSI, has been performed. Such studies require access to data with many cancer-treated men and an adequate follow-up regarding their reproductive life.

According to the recently launched European Atlas of Fertility Treatment Policies (‘Atlas of fertility treatment policies in Europe—Fertility Europe’, 2021), Sweden has been classified as ‘very good’ when it comes to providing access to a well-funded, fertility treatment evenly across the country. Therefore, we could assume that since it is nearly independent of socioeconomic status, ART in Sweden is provided to the majority of male cancer survivors with a desire for fatherhood but with the inability to conceive a child naturally.

The objective of this study was, using national Swedish population-based registries, to estimate the proportion of ART-conceived children among male cancer survivors, and to identify diagnostic subgroups most likely to conceive their child using ART.

## Materials and methods

### Data extraction from national registers

The cohort was created by combining information from the Swedish Medical Birth Register, the Swedish Multi-generation Register, the Swedish Register of Education, the Swedish Cancer Register, and the Swedish National Quality Register of Assisted Reproduction (Q-IVF). Ethical approval was granted by the ethics committee at Lund University (LU 2016/104).

**Figure 1.**
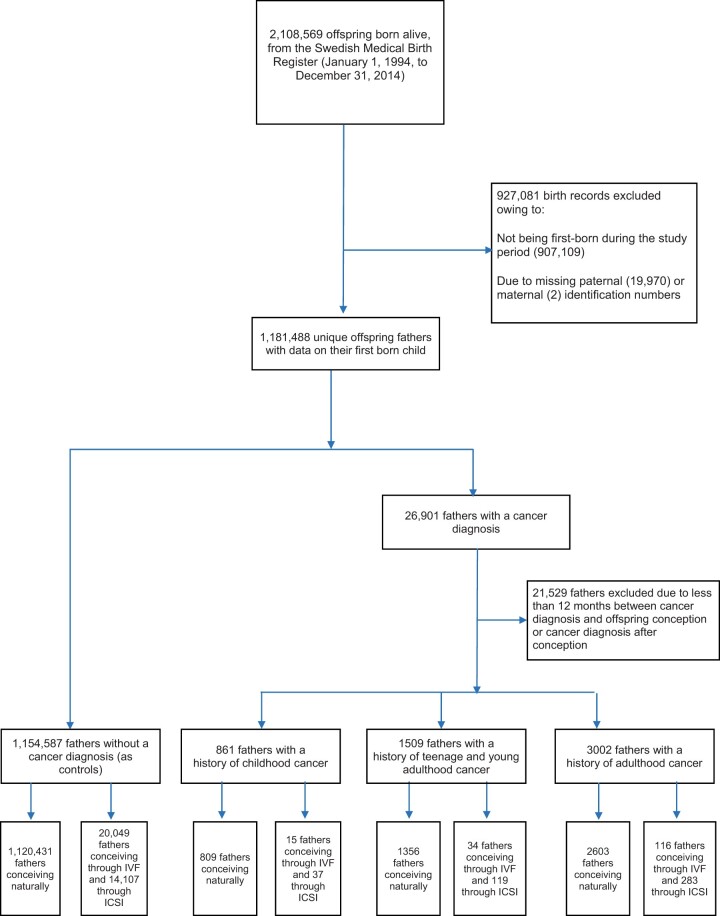
**Flowchart of inclusion process and linkage to the nationwide registers**.

Information on all children born alive in Sweden between 1994 and 2014 (2 108 569) and their fathers, was retrieved from the Swedish Medical Birth Register and the Swedish Multi-generation Register, respectively. Subsequently, non-first born offspring during the study period (907 109) and cases of missing paternal (19 970) or maternal (2) identification numbers, were excluded. Furthermore, using data on gestational length from the Swedish Medical Birth Register, we could estimate dates of conception. Among fathers with a cancer diagnosis, in order to ensure that the malignancy was diagnosed prior to conception, we excluded men who were diagnosed with cancer after or <12 months before offspring conception, which resulted in 1 159 959 fathers and the same number of children being included in the present analysis. The Swedish Medical Birth Register provided data on the mode of offspring conception for live births before 2007, and Q-IVF for those born in 2007 or later. The Swedish Cancer Register and the Swedish Register of Education provided data on any possible cancer diagnoses and information concerning paternal education, respectively. Information regarding any cancer diagnoses was available from 1 January 1958 until 31 December 2014 (Fig. 1).

### Paternal cancer groups

Based on the age at cancer diagnosis, cancer patients included in the cohort were grouped into three main groups: (i) childhood cancer survivors (diagnosis of cancer at age <15), (ii) teenage and young adult cancer survivors (diagnosis of cancer at age ≥15 and <24), and (iii) adult cancer survivors (diagnosis of cancer at age ≥24).

With the aim of subgroup analyses, fathers with a history of cancer were further divided into eight categories based on the information regarding cancer localization. The groups were defined according to the *International Classification of Diseases, 7th revision* (ICD-7), and were as follows: (i) skin cancer (ICD-7: 140.0–140.9, 190.0–191.9); (ii) prostate cancer (ICD-7: 177.0–177.9); (iii) testicular cancer (ICD-7: 178.0–178.9); (iv) digestive, respiratory, and urogenital tract cancers (ICD-7: 141.0–163.9, 179.0–181.9); (v) central nervous system and eye cancers (ICD-7: 192.0–193.1); (vi) soft tissue and bone cancers (ICD-7: 193.3, 193.8, 193.9, 196.0–197.9); (vii) hematological and lymphatic cancers (ICD-7: 200.0–207.9); and (viii) all other cancer diagnoses (ICD-7: 164.0–164.9, 170.1, 170.2, 194.0–194.9, 195.0–195.9, 199.1–199.9).

### Statistical analysis

Associations between age at onset of cancer, as well as type of cancer diagnosis, and conception through either ICSI or IVF were evaluated using univariate and multivariate logistic regression models, which yielded an unadjusted and adjusted odds ratio (aOR) with a 95% CI. Information on use of donated spermatozoa was only available from Q-IVF, and thus, the analysis of likelihood of fathering a child through ART with donated gametes was restricted to children born between 2007 and 2014. The length of the time interval since completion of cancer treatment could affect the utilization of ART, both due to the degree of recovery of spermatogenesis but also other factors, including the number of attempts to achieve pregnancy and age-related changes in fertility of the partner. Therefore, we also examined the association between the number of years between cancer diagnosis and offspring conception (1–3, 3–5, and >5) and likelihood of using ART, when compared to controls. This analysis was restricted to only adult cancer survivors and controls were matched on year of childbirth, with a 1:1 ratio. Variables used in the adjusted model were fathers’ age at childbirth (continuous) and paternal years of formal education (<10, 10–14, ≥15, or missing data), factors known to be associated with use of assisted reproduction. The first case of successful birth through ICSI treatment was reported in 1992 (Van Steirteghem, 2012), which is why access to this type of ART was limited in the beginning of the study period. Therefore, we also decided to adjust for the year of offspring conception (continuous) in the adjusted model. Fathers without a cancer diagnosis were used as controls in all analyses performed.

A *P*-value <0.05 (two-sided) was considered as statistically significant. All statistical analyses were conducted using IBM SPSS Statistics version 28 (IBM Corp.).

## Results

### Study population

Among a total of 1 159 959 fathers included in the cohort, 1 125 431 (97%), 20 220 (1.7%), and 14 560 (1.3%) of their first-born children were conceived naturally, or through IVF and through ICSI, respectively. Among fathers with history of cancer, 861 (16.0%) fathers were diagnosed with childhood cancer, 1509 (28.1%) with teenage and young adulthood cancer, and 3002 (55.9%) with adult cancer. Among childhood cancer survivors, 6.0% conceived by ART, while this proportion was 10.1% for teenage and young adult cancer survivors, 13.3% for the adult cancer survivors, and 3.0% for the controls. Further background characteristics of the fathers are shown in Table I.

**Table I:** Characteristics of all fathers included in the cohort.

	Fathers with history of childhood cancer (n = 861)	Fathers with history of teenage or young adult cancer (n = 1509)	Fathers with a history of adult cancer (n = 3002)	Fathers with no cancer diagnosis (n = 1 154 587)
**Cancer site**				
Skin	15 (1.7%)	192 (12.7%)	628 (20.9%)	—
Testicle	37 (4.3%)	359 (23.8%)	870 (29.0%)	—
Prostate	0 (0%)	0 (0%)	25 (0.8%)	—
Digestive, respiratory, or urogenital tract	124 (14.4%)	192 (12.7%)	482 (16.0%)	—
CNS or eye	221 (25.7%)	186 (12.3%)	241 (8.0%)	—
Soft tissue or bone	105 (12.2%)	138 (9.2%)	116 (3.9%)	—
Hematological or lymphatic system	321 (37.3%)	335 (22.2%)	404 (13.5%)	—
Other	38 (4.4%)	107 (7.1%)	236 (7.9%)	—
**Mean (SD) time between cancer diagnosis and offspring conception, years**	22.8 (7.29)	10.9 (5.92)	5.74 (4.33)	—
**Mean (SD) age at cancer diagnosis, years**	6.83 (4.76)	19.8 (2.49)	31.4 (6.88)	—
**Mean (SD) age at offspring birth, years**	28.3 (5.02)	29.1 (4.56)	32.3 (4.75)	28.9 (5.20)
**Years of formal education**				
<10	92 (10.7%)	134 (8.9%)	287 (9.6%)	138 230 (12%)
10–14	486 (56.4%)	873 (57.9%)	1522 (50.7%)	652 491 (56.5%)
≥15	281 (32.6%)	500 (33.1%)	1185 (39.5%)	353 721 (30.6%)
Missing data	2 (<1%)	2 (<1%)	8 (<1%)	10 145 (<1%)

CNS: central nervous system.

### Conceiving through IVF and ICSI in male cancer survivors

When compared to controls, the entire group of cancer survivors was statistically significantly more likely to father through ART (aOR 3.18, 95% CI 2.91–3.48; *P* < 0.001). Adult cancer survivors were more likely to father through both ICSI (aOR 5.52, 95% CI 4.86–6.27; *P* < 0.001) and IVF treatment (aOR 1.32, 95% CI 1.09–1.60; *P* = 0.004), while teenage and young adult cancer survivors only had a statistically significant increased likelihood of conceiving through ICSI (aOR 6.84, 95% CI 5.64–8.30; *P* < 0.001) and not IVF treatment (aOR 1.27, 95% CI 0.90–1.80; *P* = 0.17). Similarly, childhood cancer survivors were only more likely to conceive a child through ICSI (aOR 3.52, 95% CI 2.52–4.93; *P* < 0.001) but not through IVF (aOR 1.02, 95% CI 0.61–1.70; *P* = 0.955).

When compared to the general population, childhood cancer survivors (aOR 8.84, 95% CI 4.41–17.7; *P* < 0.001), teenage and young adult cancer survivors (aOR 8.28, 95% CI 5.02–13.6; *P* < 0.001), and adult cancer survivors (aOR 5.46, 95% CI 3.78–7.90; *P* < 0.001) were all more likely to father a child through ART using donated spermatozoa. The proportions of men utilizing donated spermatozoa for conception of their offspring were 33.3%, 7.6%, and 8.3% for childhood, teenage and young adulthood, and adult cancer groups, respectively.

The adjusted ORs for ICSI conception according to the cancer diagnosis are given in Table II. Childhood survivors of testicular cancer (aOR 5.15, 95% CI 1.20–22.0; *P* = 0.027), soft tissue and bone cancers (aOR 4.70, 2.13–10.40; *P* < 0.001), hematological and lymphatic cancers (aOR 4.49, 95% CI 2.72–7.40; *P* < 0.001), or central nervous system (CNS) and eye cancers (aOR 2.64, 95% CI 1.23–5.67; *P* = 0.012), were at an increased likelihood of fathering through ICSI. Teenage and young adult survivors of testicular cancer (aOR 15.4, 95% CI 11.5–20.7; *P* < 0.001), hematological and lymphatic cancers (aOR 9.84, 95% CI 6.93–14.0; *P* < 0.001), or soft tissue and bone cancers (aOR 6.83, 95% CI 3.53–13.2; *P* < 0.001) were more likely to father through ICSI treatment. Adult survivors of prostate cancer (aOR 15.7, 95% CI 6.70–36.9; *P* < 0.001), testicular cancer (aOR 9.54, 95% CI 7.81–11.7; *P* < 0.001), hematological and lymphatic cancers (aOR 11.3, 95% CI 8.63–14.9; *P* < 0.001), digestive, respiratory, and urogenital tract cancers (aOR 2.62, 95% CI 1.75–3.92; *P* < 0.001), CNS and eye cancers (aOR 2.74, 95% CI 1.48–5.08; *P* = 0.001), or skin cancer (aOR 1.68, 95% CI 1.08–2.62; *P* = 0.022) were more likely to father through ICSI treatment. The adjusted ORs for IVF conception according to the cancer diagnosis are also given in Table II. Among teenage and young adult cancer survivors, a history of testicular cancer (aOR 1.77, 95% CI 0.98–3.18; *P* = 0.057) and hematological and lymphatic cancers (aOR 1.98, 95% CI 1.10–3.56; *P* = 0.022) was associated with an increased likelihood of achieving paternity through IVF treatment. Adult survivors of testicular cancer (aOR 1.88, 95% CI 1.37–2.58; *P* < 0.001) and hematological and lymphatic cancers (aOR 1.53, 95% CI 0.94–2.47; *P* = 0.088) (Table II), were more likely to conceive through IVF, compared to the background population. No cancer types among childhood cancer survivors were shown to be significantly associated with fathering through IVF treatment (Table II).

**Table II:** Likelihood of fathering through ICSI and IVF, according to the localization of cancer in childhood, teenage, young adult, and adult cancer survivors.

Cancer group	Fathering through ICSI in cancer survivors compared to non-cancer patients				Fathering through IVF in cancer survivors compared to non-cancer patients			
	Unadjusted model		Adjusted model		Unadjusted model		Adjusted model	
	Odds ratio (95% CI)	*P*-value	Odds ratio (95% CI)	*P*-value	Odds ratio (95% CI)	*P*-value	Odds ratio (95% CI)	*P*-value
**Childhood cancer survivors**								
Skin cancer	—	—	—	—	—	—	—	—
Testicular cancer	4.62 (1.11–19.2)	0.035	5.15 (1.20–22.0)	0.027	1.57 (0.22–11.5)	0.656	1.74 (0.23–13.0)	0.591
Digestive, respiratory, and urogenital tract cancers^‡^	1.33 (0.33–5.36)	0.693	1.26 (0.31–5.17)	0.75	0.46 (0.06–3.29)	0.439	0.44 (0.06–3.20)	0.418
CNS and eye cancers	2.64 (1.25–5.62)	0.011	2.64 (1.23–5.67)	0.012	1.31 (0.54–3.18)	0.551	1.38 (0.56–3.39)	0.479
Soft tissue and bone cancers	5.78 (2.68–12.4)	<0.001	4.70 (2.13–10.4)	<0.001	1.10 (0.27–4.45)	0.895	0.89 (0.22–3.68)	0.872
Hematological and lymphatic cancers	4.52 (2.77–7.37)	<0.001	4.49 (2.72–7.40)	<0.001	0.90 (0.37–2.17)	0.806	0.96 (0.39–2.34)	0.925
All other cancer diagnoses	4.49 (1.08–18.7)	0.039	4.45 (1.03–19.3)	0.046	1.53 (0.21–11.1)	0.675	1.52 (0.20–11.4)	0.687
**Teenage and young adult cancer survivors**								
Skin cancer	0.85 (0.21–3.43)	0.82	0.79 (0.20–3.20)	0.74	0.90 (0.29–2.81)	0.854	0.82 (0.26–2.60)	0.74
Testicular cancer	16.2 (12.3–21.4)	<0.001	15.4 (11.5–20.7)	<0.001	1.96 (1.10–3.48)	0.022	1.77 (0.98–3.18)	0.057
Digestive, respiratory, and urogenital tract cancers^‡^	2.16 (0.89–5.26)	0.089	2.30 (0.94–5.68)	0.07	1.51 (0.62–3.68)	0.361	1.60 (0.65–3.95)	0.307
CNS and eye cancers	1.33 (0.42–4.15)	0.628	1.48 (0.47–4.65)	0.506	0.31 (0.04–2.18)	0.237	0.37 (0.05–2.61)	0.315
Soft tissue and bone cancers	6.32 (3.32–12.0)	<0.001	6.83 (3.53–13.2)	<0.001	—	—	—	—
Hematological and lymphatic cancers	10.3 (7.38–14.5)	<0.001	9.84 (6.93–14.0)	<0.001	2.10 (1.18–3.74)	0.012	1.98 (1.10–3.56)	0.022
All other cancer diagnoses	0.76 (0.11–5.47)	0.787	0.72 (0.10–5.23)	0.749	0.53 (0.08–3.83)	0.532	0.52 (0.07–3.76)	0.516
**Adult cancer survivors**								
Skin cancer	2.80 (1.81–4.32)	<0.001	1.68 (1.08–2.62)	0.022	2.44 (1.65–3.62)	<0.001	1.34 (0.90–2.01)	0.151
Testicular cancer	13.1 (10.8–15.8)	<0.001	9.54 (7.81–11.7)	<0.001	2.87 (2.11–3.92)	<0.001	1.88 (1.37–2.58)	<0.001
Prostate cancer	45.5 (20.1–103)	<0.001	15.7 (6.70–36.9)	<0.001	2.36 (0.32–17.4)	0.401	0.69 (0.09–5.17)	0.717
Digestive, respiratory, and urogenital tract cancers^‡^	4.61 (3.10–6.85)	<0.001	2.62 (1.75–3.92)	<0.001	1.57 (0.90–2.72)	0.109	0.79 (0.46–1.39)	0.419
CNS and eye cancers	3.87 (2.11–7.08)	<0.001	2.74 (1.48–5.08)	0.001	0.96 (0.36–2.57)	0.927	0.62 (0.23–1.68)	0.346
Soft tissue and bone cancers	2.89 (1.07–7.83)	0.037	2.16 (0.79–5.93)	0.135	0.99 (0.25–4.02)	0.992	0.67 (0.17–2.76)	0.583
Hematological and lymphatic cancers	16.7 (12.8–21.6)	<0.001	11.3 (8.63–14.9)	<0.001	2.64 (1.65–4.23)	<0.001	1.53 (0.94–2.47)	0.088
All other cancer diagnoses	8.31 (5.36–12.9)	<0.001	5.21 (3.30–8.21)	<0.001	2.50 (1.33–4.72)	0.005	1.36 (0.71–2.61)	0.351

Additional covariates in adjusted model: fathers’ age at childbirth, paternal years of formal education, and year of offspring conception.

Missing columns signifies lack of data for a statistical analysis.

‡ Excluding testicular and prostate cancer.

CNS: central nervous system.

The likelihood of fathering a child through ICSI in adult cancer survivors conceiving 1–3 years (aOR 4.55, 95% CI 3.16–6.56; *P* < 0.001), 3–5 years (aOR 3.75, 95% CI 2.49–5.66; *P* < 0.001) and more than 5 years (aOR 6.31, 95% CI 4.49–8.87; *P* < 0.001) after cancer diagnosis was approximately equally elevated. However, the aORs for achieving paternity through IVF in adult cancer survivors was only slightly elevated during all three time periods, with this increase reaching level of statistical significance only for those becoming fathers 3–5 years post-cancer diagnosis (Table III).

**Table III:** Likelihood of fathering through ICSI or IVF for adult cancer survivors, according to years between cancer diagnosis and offspring conception.

	Fathering through ICSI in adult cancer survivors compared to non-cancer patients				Fathering through IVF in adult cancer survivors compared to non-cancer patients			
Years between cancer diagnosis and offspring conception	Unadjusted model		Adjusted model		Unadjusted model		Adjusted model	
	Odds ratio (95% CI)	*P*-value	Odds ratio (95% CI)	*P*-value	Odds ratio (95% CI)	*P*-value	Odds ratio (95% CI)	*P*-value
1–3	5.21 (3.64–7.47)	<0.001	4.55 (3.16–6.56)	<0.001	1.54 (0.97–2.43)	0.065	1.14 (0.72–1.82)	0.582
3–5	4.23 (2.83–6.33)	<0.001	3.75 (2.49–5.66)	<0.001	2.22 (1.41–3.49)	<0.001	1.60 (1.01–2.54)	0.047
>5	7.64 (5.53–10.6)	<0.001	6.31 (4.49–8.87)	<0.001	2.40 (1.66–3.48)	<0.001	1.45 (0.98–2.15)	0.061

Additional covariates in adjusted model: fathers’ age at childbirth, paternal years of formal education, and year of offspring conception.

## Discussion

In this study, we found that, as compared to fathers without history of cancer, a statistically significant higher proportion of male cancer survivors achieve paternity through ICSI treatment. Offspring conception through IVF was slightly, but statistically significant increased, among adult but not among teenage and young adult, or childhood male cancer survivors. Our findings are in accordance with previously published data which implies that young male cancer survivors are at a 3-fold increased risk of utilizing ART (Gunnes et al., 2016). A Norwegian study found that the probability of first-time paternity at the age of 35 years was of the same magnitude in male cancer survivors as in men from the general population (Magelssen *et al.*, 2008). However, the reported post-treatment hazard ratio of paternity in males treated for cancer was decreased in another report (Cvancarova *et al.*, 2009).

In Sweden, reduced male fertility is still the major indication for undergoing ICSI treatment (Hamberger *et al.*, 1998). Thus, in our study, undergoing ICSI treatment can be used as a proxy for severe male subfertility, and higher utilization of ICSI within a patient group can be attributed to more severe impairment of male fertility.

It is by law mandatory to report all newly diagnosed cancers to the Swedish Cancer Register, which thus has a completeness of 96% (Barlow *et al.*, 2009). Similar rules apply to the remaining registers applied in this study, hence ensuring a near complete assessment of patient records (Axelsson, 2003; Ekbom, 2011). Information about conception through intrauterine insemination, which is a rarely used assisted reproduction technique in Sweden, was not available for the entire cohort. Therefore, for the purpose of this study, ART only refers to conception through either IVF or ICSI.

As compared to controls, all three groups of cancer survivors were more likely to conceive through ICSI. The probability of using IVF was statistically significantly increased for the adult group, with the risk estimate being similar for teenage and young adult cancer survivors, without reaching the level of statistical significance. No increase in IVF use was seen for those diagnosed with childhood cancer. This finding could be due to more severe forms of infertility in the latter group as compared to those treated during adulthood. This theory is further supported when considering that childhood cancer survivors was the cancer group with the highest utilization of donated spermatozoa, which could be used as a proxy for azoospermia.

From the point of view of planning for future offspring, our results imply that adult cancer survivors can be informed that their likelihood of requiring ICSI to achieve paternity is of approximately the same magnitude, regardless of the time since completion of cancer treatment.

In Sweden, cryopreservation of semen prior to cancer treatment is widely used. In cases where the cancer treatment has led to permanent sterility, cryopreserved gametes can be used for fertility treatment, with the preferred method being ICSI. Therefore, the higher rates of ICSI use among almost all cancer groups could be partly attributed to the use of sperm cryopreserved prior to gonadotoxic treatment. However, as has been previously shown, only a minority of male cancer survivors decide to use their cryopreserved spermatozoa (Ferrari *et al.*, 2016), and this is usually done in cases of post-cancer azoospermia or severe oligozoospermia. Therefore, a lack of information on use of cryopreserved gametes has limited the impact of the estimates regarding consumption of ART in this cohort.

As expected, men diagnosed with testicular and prostate cancer were substantially more likely to conceive through ART, when compared to the own fathers, as these malignancies directly affect the reproductive system. For patients diagnosed with cancer, a reduced fertility status caused by the cancer itself, even before any cancer therapy, has been observed by numerous studies (Gandini *et al.*, 2003; Huyghe *et al.*, 2004; Auger *et al.*, 2016; MacKenna *et al.*, 2017). This effect is most noticeable in men with testicular cancer. This is suspected as being due to not only its localization but also may be related to testicular dysgenesis syndrome, where the development of testicular germ cell cancer and decreased testicular function are pathogenetically linked (Skakkebaek *et al.*, 2001). Both orchidectomy as well as the cytostatic and irradiation regimes have been shown to significantly contribute to the reduced semen quality in survivors of testicular cancer (Huyghe *et al.*, 2004; Sprauten *et al.*, 2014). However, it cannot be disregarded that sexual dysfunction could also contribute to the inability to conceive a child spontaneously.

Prostatectomy is usually applied in the treatment of prostate cancer and leads to anejaculation. In such men, post-cancer treatment gametes can be obtained using testicular sperm extraction (TESE) (Tran *et al.*, 2015). TESE retrieved gametes can only be used for fertilization through ICSI treatment which might explain why fathers with history of prostate cancer were statistically more likely to utilize ICSI but not IVF.

Our data showed that paternity rates achieved by use of ART in survivors of hematological and lymphatic cancers was similar to those in survivors treated for malignancies in reproductive organs. It is reported that the mixed cytostatic cancer therapies, especially the ones used in advanced stages of hematologic malignancies, have a particularly negative impact on gonadal function (Øvlisen *et al.*, 2021; Elenkov and Giwercman, 2022). However, some studies have indicated decreased fertility in those patients already before initiation of cancer therapy (Hallak *et al.*, 2000; Gandini *et al.*, 2003; Auger *et al.*, 2016), thus it is unclear as to what extent the impairment of testicular function is related to the chemotherapy and/or radiotherapy applied in treating those men. Another pathogenic mechanism which could contribute to a higher utilization of ART in such men is sexual dysfunction, which is estimated to affect between 20% and 54% of lymphoma survivors (Arden-Close *et al.*, 2011). This could be related to a higher prevalence of psychosocial disorders following cancer diagnosis, but also may be partially linked to the use of chemotherapy leading to impairment of testosterone production, and thereby, to a diminished libido and reduced sexual function (Greenfield *et al.*, 2007; Kiserud et al., 2009).

Our study provides further insight into which groups of patients could post-treatment be the most likely to use ART. Such information is useful for physicians and other healthcare workers for the purpose of counseling young male cancer survivors concerning the risk of infertility, and even for policy makers in order to provide equal access to fertility treatment. Accessibility to ART across Europe varies with only 3 out of 43 countries (7.0%) offering full funding for up to 6 cycles of IVF/ICSI treatment (‘Atlas of fertility treatment policies in Europe—Fertility Europe’). Income level has been linked to incidence rates of many common cancers (Larsen *et al.*, 2020; Lortet-Tieulent *et al.*, 2020), and thus the population with a lower socioeconomic status is more likely to struggle financially with the inability to afford expensive fertility treatments, if they suffer from cancer-treatment induced subfertility. In this sense, in countries with no or poor funding of ART, the social status of the cancer survivors might be a main determining factor in family planning. Our results demonstrate that ever since the implementation of ART in Sweden, 6% of childhood cancer survivors and 13% of adult cancer survivor who fathered a child, needed to use ART to produce offspring after cancer treatment. Our findings would be useful for targeting populations which could mostly benefit from improvements in public funding and/or lowered costs of ART, when improving fertility treatment policies.

Furthermore, the finding of almost all categories of male cancer survivors being more likely to father through using ART, underlines the need for developing new cancer therapies with lower gonadotoxicity. Maintaining fertility or achieving paternity after being cured for cancer is a major contributor to sustained quality of life in those men (Klosky et al., 2015; Patterson *et al.*, 2021), and as the number of cancer survivors will keep increasing, the importance of this issue will be growing.

A major strength of this study is the use of nationwide Swedish registers, which resulted in the inclusion of over 1.1 million men in the cohort. Moreover, as mentioned earlier, owing to the high completeness of the compulsory datasets (Axelsson, 2003; Barlow *et al.*, 2009; Ekbom, 2011), information on use of ART in practically all Swedish male cancer survivors was available. However, inclusion of only livebirths can be considered as a limitation since the cases where the pregnancy has ended in miscarriage or stillbirth are not reported. Furthermore, the most severe cases of male infertility could have been missed since there is a lack of data on men for whom ART was not applied or was not successful. Hence no definite conclusions can be made concerning the extent of infertility among different groups of cancer survivors. Additionally, some severe cases of male infertility might have been managed by insemination with donated spermatozoa which would have led to those men being categorized as conceiving naturally. However, such misclassifications would more likely strengthen than weaken the statistically significant associations reported by us. Another weakness is the lack of treatment data, which invalidates our ability to provide risk estimates adjusted for the intensity and type of the cancer treatment given. However, even such a refinement of our analyses would not eliminate the need for individualized counseling even taking into consideration other factors, such as the pre-treatment fertility status of the patient.

In conclusion, men with a history of cancer had two to three times higher odds of using of IVF/ICSI treatments to father a child, when compared to those not being treated for cancer. Although almost all male cancer survivors were more likely to use ART, patients treated for malignancies in male reproductive organs or in hematological or lymphatic system, particularly those diagnosed during teenage years or young adulthood and later, were most likely to conceive through ART. Information concerning the potential post-cancer fertility treatment, fertility issues, and fertility preservations options should be made easily accessible for male cancer patients.

## Data Availability

If conditions are met under Swedish Law, researchers can obtain anonymized data by contacting the corresponding author.
